# Vasotocin influences mother–offspring associations in a facultatively family-living lizard

**DOI:** 10.1093/beheco/arag082

**Published:** 2026-07-21

**Authors:** Jai Lake, Geoffrey M While, Alexander G Ophir, Dustin R Rubenstein, Lynea Witczak Oldfather, Martin J Whiting

**Affiliations:** School of Natural Sciences, Macquarie University, Sydney, New South Wales 2109, Australia; School of Natural Sciences, University of Tasmania, Hobart, Tasmania 7001, Australia; Department of Psychology, Cornell University, Ithaca, NY 14853, United States; Department of Ecology, Evolution and Environmental Biology, Columbia University, New York, NY 10027, United States; Department of Biology, Elon University, Elon, NC 27244, United States; School of Natural Sciences, Macquarie University, Sydney, New South Wales 2109, Australia

**Keywords:** parental behavior, aggression, vasotocin, *Liopholis whitii*, social behavior

## Abstract

Parent–offspring associations provide the basis for more complex care and breeding systems. One proximate mechanism by which parental behavior is facilitated across animal taxa is via nonapeptide hormones such as arginine vasotocin (AVT). However, existing research largely comes from obligately social species, and whether such mechanisms are conserved in social systems where care is facultative remains unclear. Here, we used a facultatively family living skink, *Liopholis whitii*, to test the role of AVT in mediating 2 key parental behaviors: parent–offspring associations (measured by proximity between mothers and offspring) and maternal aggression (in response to a conspecific model). We found that increasing AVT via intraperitoneal injection led to more time spent in closer proximity between mother and offspring. Conversely, a decrease in AVT signaling (using an AVT receptor antagonist) resulted in a reduction in the time spent in closer proximity. We also found that these effects caused by increased AVT signaling persisted for several weeks, suggesting a potential organizational effect of AVT on the mother–offspring relationship. Contrary to our predictions, we found no effect of AVT manipulations on maternal aggression, suggesting that AVT may have been de-coupled from its usual role as a moderator of aggression. These results support the idea that AVT may be a mechanism involved in governing parental behavior in this system, and as such, could have played a key role in the repeated evolution of group living.

## Teaser

Although vasotocin (AVT) is a hormone that influences parental behavior in numerous animal species, until now it has never been investigated in lizards. In White’s skinks, experimentally increasing or decreasing AVT activity has corresponding effects on the amount of time that a mother spends in proximity to her offspring, and these effects persist for several weeks. However, AVT does not affect territorial defense behaviors, despite the importance of this in the context of parental care.

## Introduction

Parental behavior, defined here as any post-fertilization behavior that increases offspring survival or fitness (Royle et al. 2012), has evolved repeatedly and independently and is likely a precursor to more complicated social behavior such as monogamy and cooperative breeding (Freeman and Young 2013; Socias-Martínez and Kappeler 2019). Across taxa, the bedrock of parental behavior is an association between parent and offspring. These associations can range from simple maternal tolerance of offspring such as that observed in lizards (Chapple and Keogh 2006; While et al. 2009) to the multiyear investments made in offspring by our own species. The taxonomic breadth and diversity in parental behavior has led to a significant research effort investigating both the ultimate and proximate mechanisms responsible for its repeated evolution (Royle et al. 2012).

The nonapeptide hormone arginine vasotocin (AVT—often referred to as vasopressin in mammals; but see Theofanopoulou et al. 2021) is a signaling molecule that serves as a proximate mechanism underlying nearly all aspects of vertebrate social behavior (Goodson and Thompson 2010; Bosch and Neumann 2012; Choleris et al. 2013; Wilczynski et al. 2017), including parent–offspring associations (Zimmermann-Peruzatto et al. 2015; Feldman and Bakermans-Kranenburg 2017). For example, increased AVT activity, either through natural variation in receptor density or experimental supplementation, leads to more grooming, nursing, and pup-carrying in rats, mice, and voles, while AVT antagonists inhibit these same behaviors (Pedersen et al. 1982; Wang et al. 1994; Bosch and Neumann 2008; Curley et al. 2012). In marmosets, increases of AVT lead to greater responsiveness to infant cues (Taylor and French 2015), and specific differences in the promotor region of the human vasotocin receptor gene are also linked to maternal behavior (Avinun et al. 2012). In zebra finches, blocking AVT in prospective parents reduces nest-building behavior (Klatt and Goodson 2013), and biparental cichlid fish have greater AVT activity during nest construction (Cunha-Saraiva et al. 2019). Conversely, in the poison frog, *Ranitomeya imitator*, AVT supplementation decreases egg attendance (Schulte and Summers 2017). In the only reptile study to investigate the role AVT plays in parental behavior, Lind et al. (2017) found that blocking AVT disrupts mother–offspring association in a viviparous rattlesnake, *Sistrurus miliarius* (Lind et al. 2017). Together, this research suggests that relatively small changes in AVT can underly and influence a range of parental behaviors, even over relatively long time periods (eg, days to weeks), either via downstream actions on other hormones (eg, Meylan et al. 2017) or through organizational effects on the brain during sensitive developmental periods (eg, Baran et al. 2016).

Most previous studies have largely focused on exploring the role that AVT plays in mediating the nature and extent of parental behavior in systems where care is complex and obligate. We argue that AVT may also play a role in the emergence of parental behavior. In these systems, parental behavior is represented by an increase in the extent of social association (or grouping) between parents and offspring. Crucially, AVT has been shown to facilitate social grouping in vertebrates. For example, blocking AVT receptors in normally gregarious finches decreased preference to spend time in larger groups (Kelly et al. 2011), and AVT supplementation elicits positive phonotaxis in frogs (Baugh and Ryan 2017). It is therefore plausible that AVT initially played a key role in mediating these early parent–offspring interactions before being co-opted to regulate the more complex forms of parental behavior seen in species with derived care. To test this idea, we need to explore the role that AVT plays in mediating simple parent–offspring grouping in systems where parental behavior is nonobligate (ie, the offspring’s survival is not dependent on the presence of a parent). Examining the extent to which AVT underpins parental behavior in such systems will ultimately provide a broader view of the mechanistic control of these behaviors.

White’s skinks (*Liopholis whitii*) are a social (family-living) lizard species that provide an excellent opportunity to test these ideas. Family life in these lizards is underpinned by prolonged associations between parents and offspring. However, these associations are facultative, with some offspring remaining in their parents’ territory for the first year or 2 of life, whereas others disperse immediately (Chapple and Keogh 2006; While et al. 2009). Whether offspring disperse or not varies both within and between populations and is positively associated with constraints on habitat availability (While et al. 2009; Botterill-James et al. 2016). This form of parental behavior is relatively simple and can be defined as tolerance to offspring proximity. Although parents whose offspring remain do not engage in any obvious active parental care behaviors, such as provisioning, by remaining in close proximity to their parents, young *L. whitii* benefit from the territorial behavior of their parents, which serves as indirect protection from potentially cannibalistic conspecifics (Sinn et al. 2008). The flexible parental strategy seen in *L. whitii* makes it an excellent study species in which to investigate the mechanisms mediating simple parental behavior.

Here, we tested whether AVT plays a role in mediating variation in parental behavior in *L. whitii*. We began with a targeted experimental approach in which we artificially facilitated or inhibited AVT action in mothers and measured their proximity to their offspring over the short-term (ie, hours) and long-term (ie, weeks). We then performed simulated territorial intrusions to measure the effects of AVT on protective maternal aggression. We predicted that AVT administration would result in increased mother–offspring proximity and greater maternal aggression toward intruders, and AVT inhibition would result in a reduction of these effects. In testing whether AVT mediates the facultative parental behavior in *L. whitii*, we aimed to elucidate the mechanisms underlying simpler and more flexible forms of parenting.

## Materials and methods

### Study species and husbandry

We collected animals under NSW government scientific license (SL101972), and the study was reviewed and approved by the Macquarie University Animal Ethics Committee (ARA 2022/022). We caught 55 gravid adult female *L. whitii* from a field site near Jindabyne, New South Wales, Australia in January 2023, by lassoing and by hand. Once captured, animals were weighed with a digital scale to the nearest 0.01 g and measured for snout-vent-length (SVL) and total length to the nearest 1 mm with a standard ruler. We transported lizards in cloth bags back to Macquarie University Wallumattagal Campus (Sydney). We housed each lizard individually in opaque plastic enclosures (415 W × 320 H × 620 L mm), which contained a single plastic shelter, cat litter substrate, and a water bowl. All terraria were in a climate-controlled room (maintained at 24 °C) illuminated by 30 W Outback Max 10 UV tubes (Ultimate Reptile Suppliers) and had heat wire running underneath one side to provide a thermal gradient (24 to 33 °C). Heating and lighting were set to a 12 h cycle starting at 07:00 to simulate conditions experienced in the wild. Lizards were fed once a week with baby food (Heinz, various flavors) and twice a week with live crickets dusted with calcium and vitamin powders (Ultimate Reptile Suppliers).

Females were checked at least once daily for births, which took place between 29 January and 23 February. In our study, litter size ranged from 1 to 4 (mean ± SD = 2.47 ± 0.63), and in cases of litters of more than 1 offspring, females gave birth over several days (mean ± SD = 2.17 ± 1.31; range = 1 to 6). Because of this asynchronous birthing, we were able to individually mark offspring in the order in which they were born using a xylene-free paint pen (Artline). We left the firstborn offspring in the mother’s enclosure but removed all subsequent offspring shortly after birth and housed them separately in sibling groups. In 6 cases when 2 or more offspring were discovered at the same time, the heaviest of the offspring was kept with the mother. In one case, the first-born offspring went missing 1 d post birth (presumed to have been cannibalized by the mother) and so the second-born offspring was kept with the mother. Offspring could not be sexed because females are born with hemipenal structures that they retain until maturity, and sexes are otherwise identical (Chapple 2006). As soon as offspring were born, they were weighed and measured following the same procedure as for adults. All surplus offspring were housed in smaller enclosures (325 W × 225 H × 470 L mm) in sibling groups, but husbandry was otherwise identical. Parturition was considered complete if mothers did not give birth to more offspring for 7 consecutive days, at which point we began behavioral trials. Mean age of the focal offspring at the start of behavioral trials was 12.5 ± 3.8 d. Variation in focal offspring age was accounted for statistically in our analysis (see Material).

At the end of the experiment, females and the offspring they were housed with were released at the mother’s point of capture. Surplus offspring were released near to their mother’s point of capture to maintain genetic population structure, while minimizing the potential for the mother to attack unfamiliar juveniles.

## Experimental procedure

### Vasotocin manipulations

We artificially increased and decreased the action of AVT via intraperitoneal injections (as in Marler et al. 1995; Propper and Dixon 1997; Burmeister et al. 2001; Semsar et al. 2001; Coddington and Moore 2003; Dunham and Wilczynski 2014; Lind et al. 2017; Campos et al. 2020). Specifically, lizards were administered AVT (AVT+), a selective AVT receptor antagonist (AVT−), or a saline control. AVT+ lizards received an injection of [Arg8]-Vasotocin (HY-P1574A, MedChem Express, Monmouth Junction, NJ, USA). AVT− lizards received an injection of [β-Mercapto-β,β-cyclopentamethylenepropionyl1, O-me-Tyr2, Arg8]-Vasopressin (V2255, Sigma-Aldrich, St. Louis, MO, USA) known commonly and referred to hereafter as Manning Compound. Manning compound selectively inhibits the AVT V1a receptor in mammals (and possibly preferentially targets V1a-like receptors in other taxa), has little diuretic activity (Kruszynski et al. 1980), and has been used in behavioral studies testing the effects of AVT in numerous taxa (Propper and Dixon 1997; Soares et al. 2012; Lind et al. 2017). Vasotocin and Manning compound were delivered in saline at 3 µg/g body mass, total injection volume equaled 10 µL/g body mass. Control group lizards received saline at 10 µL/g body mass. Lizards were restrained by hand and injected intraperitoneally with a 29-gauge needle. We waited at least 30 min between injections and observations to allow lizards to recover, during which time we monitored lizards for adverse effects. This dose and delivery method was based on similar AVT manipulations that have successfully altered behavior in reptiles and amphibians (Burmeister et al. 2001; Dunham and Wilczynski 2014; Lind et al. 2017), either through acting on peripheral targets (Campos and Belkasim 2021) or by crossing the blood–brain barrier in small quantities (Kang and Park 2000).

### Effects of AVT on mother–offspring association

To quantify how AVT influences mother–offspring associations in *L. whitii*. We randomly assigned mothers to either AVT+ (*n* = 23), AVT− (*n* = 9), or the saline control (*n* = 23) treatment. Supply chain issues prevented us from receiving enough Manning compound, leading to the smaller *n* for the AVT− group, and as such results from this treatment ought to be treated cautiously. Females in these 3 treatment groups did not differ in body mass 10 d post-parturition when controlling for SVL (ANCOVA: *F*_(2,51)_ = 1.14, *P* = 0.33). To determine whether a single AVT manipulation could have lasting effects on the mother–offspring association, we continued to monitor lizards for several weeks.

**Figure 1:**
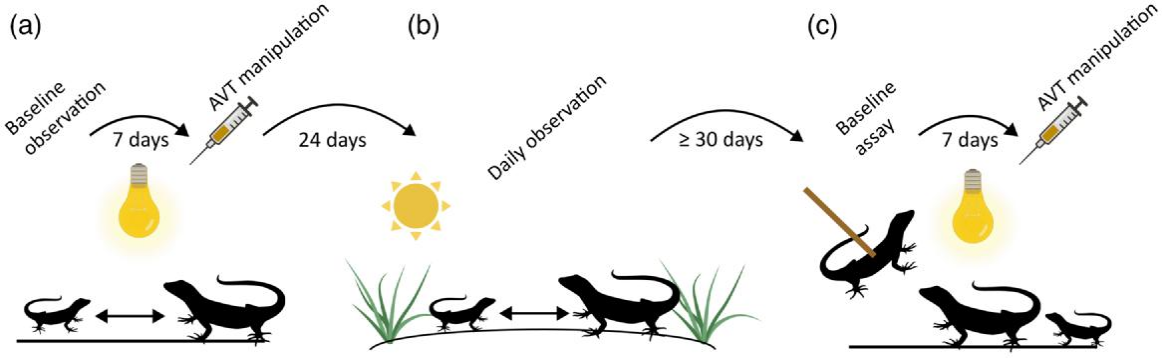
Timeline of experimental procedures. a) Seven days after the final parturition, we conducted a baseline observation to assess mother–offspring proximity. Seven days later we manipulated AVT and repeated the observation. b) 24 d after this (or 38 d after the final parturition) lizards were moved outside. Following 24 h acclimation, proximity data was collected daily for 30 d. c) When all lizards had their proximity data collected, they were moved back inside. Following 24 h acclimation, we conducted a baseline aggression assay on each lizard. Seven days later we manipulated AVT and repeated the assay. Figure created in https://BioRender.com.

We began by conducting baseline observations in which no manipulation was administered by recording the natural behavior of the mother–offspring pair in their home enclosure. Observations started the day that parturition was determined to be finished (7 d after the most recent birth). On day 14 after the final parturition, adult females were given an injection according to their treatment group, and then immediately returned to their home enclosure (Fig. 1A). Lizards were given a 30-min recovery period post-injection before recording their behavior again. In the wild, offspring typically disperse either immediately or after 1 yr (sometimes 2), with maternal tolerance remaining stable in the intervening period (While et al. 2009). As such, we did not expect the time between our baseline and post-injection observations to effect mother–offspring associations. For both the baseline and post-injection time point, the observation period lasted 70 min. All observations took place between 10:00 and 13:00 to allow lizards to reach their preferred body temperature of 34 °C (Bennett and John-Alder 1986). We video-recorded all observations with a GoPro HERO7 mounted approximately 55 cm above the enclosure.

For each video, we collected location data for each individual to estimate the amount of time they spent in close proximity to one another, defined as lizards being <20 cm apart, or approximately 1 adult body length. One body length as a measure of 2 individuals being “in proximity” has been used as a proxy for prosocial behavior (Robinson et al. 2017; Turano et al. 2024), or to characterize group dynamics and social systems in a number of species (Jenikejew et al. 2020; Hildebrandt et al. 2021; Utsumi et al. 2022), particularly in captive systems. In *L. whitii*, such maternal tolerance of offspring on her territory functions to protect the offspring from cannibalistic conspecifics (Sinn et al. 2008). To estimate the positions of each mother and offspring pair, we used Social LEAP Estimates Animal Poses (SLEAP), a software package that uses machine learning to automatically track multiple animals (Pereira et al. 2022). Videos were cropped and converted to 10 frames per second for processing. For frames where both animals were visible, an average coordinate value for their visible body parts was generated, and the distance between the 2 animals was calculated in pixels, which was then converted to cm using known pixel-cm values for each video (calculated by measuring length in pixels of an object of known size in ImageJ). Many videos, or parts of videos, contained only one animal because the other one was in a shelter or otherwise not visible. Clips in which only one animal was visible were scored using separate models that tracked adults or juveniles only, and the location of the second individual was manually inputted as the central point of whichever shelter it was taking refuge in, or as the center point of that animal if it could be partially seen but could not be detected by the software (eg, when just the snout tip was visible). Because of the constraints of SLEAP, video clips where both animals were not visible were not automatically scored, and because there were multiple potential hiding spots for the lizards (eg, in the shelter, under the water dish, buried in substrate), we did not score these manually. However, the shelter was the most common hiding spot, and so we may have underestimated time spent in proximity. The number of frames where lizards were “in proximity” or “not in proximity” was converted to proportion of time spent in proximity for the purpose of analysis, so that each video generated a single datapoint. On average, we collected data on the distance between mother and offspring from 43.7 ± 13.1 min of each 70-min video.

Previous research has found long-lasting effects after manipulating AVT (eg Boer et al. 1994; Stribley and Carter 1999; Baran et al. 2016). To test whether small changes in AVT could cause lasting shifts in behavior in *L. whitii*, we continued monitoring mother–offspring associations and space use over 30 d. To facilitate more natural space use, on day 38 after the final parturition we moved mother–offspring pairs outdoors to 1.5 m open-top enclosures contained under predator-proof netting. Each enclosure contained 2 stacks of 3 roofing tiles (23 × 38 cm) as shelters, sand substrate, and a water bowl. Lizards were given 24 h to acclimatize before data collection began, and it continued daily for 30 d (Fig. 1B). Each day, a single observer slowly approached each enclosure and recorded the position of mother and offspring relative to one another according to 8 predefined categories (see Table 1 for details). We opted for categorical scoring because lizards always moved on approach by an observer, preventing accurate straight-line measurements to be taken. Categorical scoring of relative positions could be determined from where lizards were first observed, which was usually when the observer was ∼1 m away. If lizards were not visibly basking, we slowly removed shelter tiles until each individual’s position could be determined. The order in which enclosures were checked was fixed and designed to minimize disturbance to enclosures not yet checked. Weather conditions could change over the course of data collection in a given day, and so behavior between pairs may have differed due to thermal conditions. However, since all checks were always completed in <1 h, we do not expect this to have biased the data across subjects and treatments significantly. We collapsed these 8 categories into 3 functional social categories that overlapped in terms of biological relevance: Proximity (sum of observations of shelter sharing + body contact + 1 body length), Mid-range (sum of observations of different shelter, same stack + 2 body lengths + 3 body lengths), and Distant (sum of observations of different shelter + distant) (Table 1).

**Table 1:** Relative positions of mother and offspring in semi-natural enclosures.

Position	Definition	Collapsed position
Same shelter	Both lizards are found under the same roofing tile	Proximity
Different shelter, same stack	Lizards are found under different tiles, but within the same stack of tiles	Mid-range
Different shelter	Lizards are found in 2 different stacks of tiles	Distant
Body contact	At least 1 lizard is basking, and is in body contact with the second lizard	Proximity
1 body length	At least 1 lizard is basking, and is within 1 adult body length of the other	Proximity
2 body lengths	At least 1 lizard is basking, and is within 2 adult body lengths of the other	Mid-range
3 body lengths	At least 1 lizard is basking, and is within 3 adult body lengths of the other	Mid-range
Distant	At least 1 lizard is basking, and is further than 3 body lengths from each other	Distant

Body lengths were determined visually, and equated to approximately 20 cm. Initially recorded categories were collapsed into 3 biologically relevant categories for analysis.

### Effects of AVT on protective maternal aggression

We also examined the role of vasotocin on aggressive (protective) parental behavior in *L. whitii* by quantifying the mother’s response to a territorial intrusion by a model conspecific under different pharmacological conditions. At the end of the above long-term association data collection period, we moved lizards back to indoor enclosures (described above) to control for potentially confounding factors found in the outdoor enclosures (eg, temperature, light, wind, scent, noise). Females were randomly re-assigned to 1 of the 3 treatment groups: AVT+ (*n* = 17), AVT− (*n* = 17), or control (*n* = 17). Females from each of the pharmacological treatment groups in the first part of this study were distributed evenly across the new treatment groups (Pearson’s χ2(4) = 0.87, *P* = 0.93). Nevertheless, the effect of the original treatment females received was controlled for statistically (see below). We weighed females when they were moved inside, and these weights were used to calculate doses. Females in the 3 treatment groups did not differ in body mass when controlling for SVL (ANCOVA; *F*_(2,47)_ = 0.62, *P* = 0.54). Three offspring and 1 mother died during the course of the 30-d data collection period, leaving us with 51 mother–offspring pairs for this experiment.

The assay involved simulating the intrusion of a conspecific into the territory of the focal female. The “conspecific” was a 3D-printed model skink (SVL approximately 77 mm) that was painted to appear more realistic (Figure S1) and attached to the end of a 120 cm wooden dowel with metal wire. The model was lowered slowly into the enclosure at the opposite end to the focal lizard and moved slowly across the substrate toward her, mimicking natural behavior. Subjects were touched on the snout with the tip of the model, and followed around the enclosure for up to 1 min (or until the focal lizard fled to the shelter as in Sinn et al. 2008). We collected 3 behaviors to quantify aggression, all of which are typical aggressive responses (Sinn et al. 2008): (i) the number of times the lizard bit the model; (ii) the number of times the lizard displayed an open mouth gape; and (iii) the number of times the lizard exhibited a back arch, a display in which the spine of the lizard forms a concave arch. Behaviors were scored as occurring multiple times if they were performed after each individual touch of the lizard by the model.

Testing was carried out opportunistically between approximately 11:00 and 13:10 on lizards that were basking and did not flee as the observer approached. Enclosures were arranged so that the heat cord and UV light source were at the opposite end of the enclosure to the shelter, ensuring lizards were basking in an open area, which allowed the assays to take place. Three individuals did not bask at any point for approximately 2 h, and in these cases, we gently removed their shelter and placed it at the opposite end of the enclosure, to allow the aggression assay to be carried out. We cleaned the model with 80% ethanol and air-dried it between each intrusion so as not to gather scent cues from other females. The offspring remained in the enclosure for all tests. Immediately after an assay, the mother was restrained by hand and body temperature was taken using a temperature gun held at roughly 1 to 3 cm from the ventral surface of their torso. Observations were video recorded with a GoPro HERO7 mounted above the enclosure and scored in BORIS (Friard and Gamba 2016) by a single observer (JL) blind to treatment.

We conducted an initial baseline aggression assay 24 h after the lizards had been moved inside. At the time of baseline assays, mean offspring age was 83.7 ± 5 d. We carried out a supplementary analysis using only the baseline data and found that this variation in age did not affect the mothers’ aggression (Supplementary material). One week after the baseline assays, females were given an injection according to treatment group as described above, and the aggression assay was repeated (Fig. 1C). Injections were carried out between approximately 10:35 and 11:20, and assays took place at least 40 min post-injection to allow sufficient recovery. Mean ± SD time between injection and assay was 66 ± 25 min.

### Validation of AVT supplementation

To validate both the presence and duration of an effect of our AVT treatment in *L. whitii*, we supplemented nonexperimental lizards with AVT and then sampled the concentration of hormone in plasma at 7 different time points spanning 0.5 to 48 h. We aimed to collect 5 samples for each of the 7 time points using 19 individuals, and so each lizard was sampled a maximum of 2 times, and never for the same time point. We waited a minimum of 2 wk between sampling the same lizard for the second time. We used lizards that were already housed at Macquarie University. These lizards were housed alone, but otherwise in the same conditions as described above for our experimental animals. Lizards were weighed and given a dose of AVT as described above and then returned to their home enclosure until blood sampling. Blood (<100 µL) was collected by venipuncture of the *Sinus angularis* (corner of the mouth) and was immediately placed on ice until centrifugation at 1,600 rcf for 15 min at 4 °C. Plasma was separated and stored at −80 °C until analysis. Mean ± SD time between disturbance and blood sampling was 158 ± 66.64 s. For several lizards, a sufficient volume of blood could not be collected and so only 31 samples were used for analysis.

We analyzed concentration of vasotocin (pg/mL) in plasma using an enzyme linked immunosorbent assay (ELISA; ab205928, Abcam, Melbourne, Australia). We first performed parallelism and quantitative recovery validations to ensure the ELISA kit performed similarly at different concentrations of AVT, and to decide on a suitable dilution factor for experimental plasma samples. For these validations, we used plasma pooled from 17 unmanipulated adult female *L. whitii* collected 1 yr prior to the plasma sample collection described above. Parallelism was demonstrated by an ANOVA of log optical density against log concentration of standards and serially diluted plasma samples (*F*_(2,29)_ = 2.20, *P* = 0.14). Mean ± SD quantitative recovery of AVT for *L. whitii* was 96.44 ± 29.01%. We chose a sample dilution of 1:32 based on these validations. There is disagreement in the literature regarding whether to extract plasma samples or not, with unextracted values typically leading to higher concentrations of hormone (Leng and Sabatier 2016). Extraction removes AVT that is not bound to plasma proteins, which is the majority of detectable hormone, and additionally, it is unknown whether or not bound AVT is biologically relevant (Brandtzaeg et al. 2016). Therefore, we decided not to extract our plasma samples (as in Witczak et al. 2018). Due to the limited number of plasma samples we were able to collect, we did not perform the same validations using Manning compound.

Two experimental plasma samples were removed for analysis, one of which had AVT concentrations below a detectable threshold, and one of which was suspected to be contaminated because of unusually high AVT concentrations coupled with a discoloration of the plasma. Sampling times were 0.5 (*n* = 4), 1 (*n* = 5), 2 (*n* = 4), 6 (*n* = 5), 12 (*n* = 5), 24 (*n* = 5), and 48 (*n* = 3) hours post-injection. Mean ± SD intra-assay variation was 16.3 ± 16.5%. Plotting the data suggested that vasotocin concentration was still increasing at 0.5 h post-injection. As such, to examine the rate at which vasotocin concentration dropped over time, we removed this time point from analysis. Using the data from the remaining 6 time points (1 to 48 h post-injection), we fitted a nonlinear mixed effects model using the *nlme* package in R. After correcting for our 1:32 dilution, vasotocin concentration was log-transformed for the purposes of analysis. A random intercept for ID was included to account for individual variability in baseline vasotocin concentrations.

The results we obtained from the ELISA highlight that AVT levels are increased to more than double the estimated baseline concentration in the first couple of hours post-supplementation, which corresponds to the timeframe in which we collected initial data on mother–offspring association (Figure S2). Our model showed the estimated rate of decay as −0.035 (*t* = −7.12, *P* < 0.001) suggesting a 3.5% decrease in concentration per hour, equal to an estimated half-life of 19.5 h.

### Statistical analyses

All analyses were carried out in R version 4.5.2 (R Core Team 2023). We used generalized linear mixed models (GLMMs) to assess the effect of treatment on behavior in each experiment. Treatment level *P*-values were calculated using the *Anova*() function from the *car* package (Fox and Weisberg 2019), and if a significant effect of treatment was found, we used the *emmeans* and *multcomp* packages to perform pairwise comparisons. All models were compared with corresponding null models using either Akaike’s information criterion (AIC), or a simulated likelihood ratio test (LRT). We report significance at *P* < 0.05. Plots were generated using *ggplot2* (Wickham 2016). We also conducted sensitivity analyses using G*Power (Faul et al. 2009) to determine the minimum detectable effect size, which was estimated as *f*^2^ = 0.07 and *f*^2^ = 0.08 for the short-term proximity data, and the maternal aggression trials, respectively. We calculated Cohen’s *f*^2^ as a measure of effect size for significant predictors in these models, with *f*^2^ > 0.02, *f*^2^ > 0.15, and *f*^2^ > 0.35 representing small, medium, and large effect sizes respectively (Cohen 1988). Sensitivity analysis and Cohen’s *f*^2^ calculation were not possible for the long-term proximity model due to the structure of multinomial mixed models.

### Effects of AVT on mother–offspring association

To test whether our pharmacological manipulations affected the likelihood of mother and offspring associating in the short-term, we modeled time spent in proximity as a proportion of the total observation time using the *cbind*() function (time in proximity, time not in proximity), which accounted for the different observation times between individual mother–offspring pairs. Proportion of time in proximity was fitted with a binomial distribution against pharmacological treatment and assay (baseline or post-injection), with an interaction term specified between them, using the *lme4* package (Bates et al. 2015). ID was included as a random effect, and model fit was assessed with the *DHARMa* package (Hartig 2022). To confirm there was no variation between the 3 treatment groups before the AVT manipulations, we also fitted a model using only the baseline data. This additional model also included offspring age and size to ensure these differences were not driving variation in mother–offspring association (see Table S1).

During the long-term data collection period on space use, 3 offspring and 1 mother died for reasons unrelated to the experiment, resulting in 4 pairs with fewer than 30 observation days. Two of these pairs had fewer than 10 observation days and were discarded from analysis, but we kept the other 2, which had 17 and 22 observations. We therefore had 53 mother–offspring pairs for analysis across the 3 treatment groups (AVT = 22, Manning compound = 8, control = 23). Each pair contributed 1 data point per day in a single relative position category, resulting in a total of 1,566 observations. As detailed above, we collapsed our initially recorded position categories into 3 biologically relevant ones; proximity, mid-range, and distant. To test whether our AVT treatments affected the space use of mother and offspring, we fitted a multinomial mixed-effects model using the package *mclogit* (Elff 2025). To test whether the effects of our manipulations persisted or changed across time, we included treatment and day of observation as fixed effects with an interaction. We also included ID as a random effect. The position category “distant” was set as the reference level, as this was the most commonly recorded state. We compared the multinomial model to a null model that contained the random effect only.

### Effects of AVT on protective maternal aggression

All pairs of the 3 observed behaviors were highly correlated (Bite × Gape: *r* = 0.81, *P* < 0.001; Bite × Arch: *r* = 0.68, *P* < 0.001; Arch × Gape: *r* = 0.71, *P* < 0.001) and were therefore summed so only 1 variable was being tested in subsequent analyses (hereafter referred to as “aggression response”). We analyzed differences in aggression response between the 3 treatments using a GLMM fitted with a negative binomial distribution, using the *lme4* package (Bates et al. 2015). Pharmacological treatment and assay (baseline or post-treatment) were fitted as fixed effects with an interaction. We included original pharmacological treatment, as well as mother’s body temperature and mother’s SVL (both scale-transformed), as fixed effects. ID was included as a random effect. We included the log of trial time as an offset in our model because each aggression assay was variable in the time it lasted; this was because lizards were allowed to flee and thus end the trial. Heteroscedasticity was assessed visually and tests for under/over dispersion and zero inflation were performed using the *DHARMa* package (Hartig 2022). Multicollinearity was assessed using the *vif*() function of the *car* package (Fox and Weisberg 2019). Model fit was assessed using these tests and by comparing AIC relative to identical GLMMs fitted with Poisson distribution. We also tested for repeatability in aggression response using an intraclass correlation coefficient calculated using the package *ICC* (Wolak et al. 2012).

## Results

### Effects of AVT on mother–offspring association

**Figure 2:**
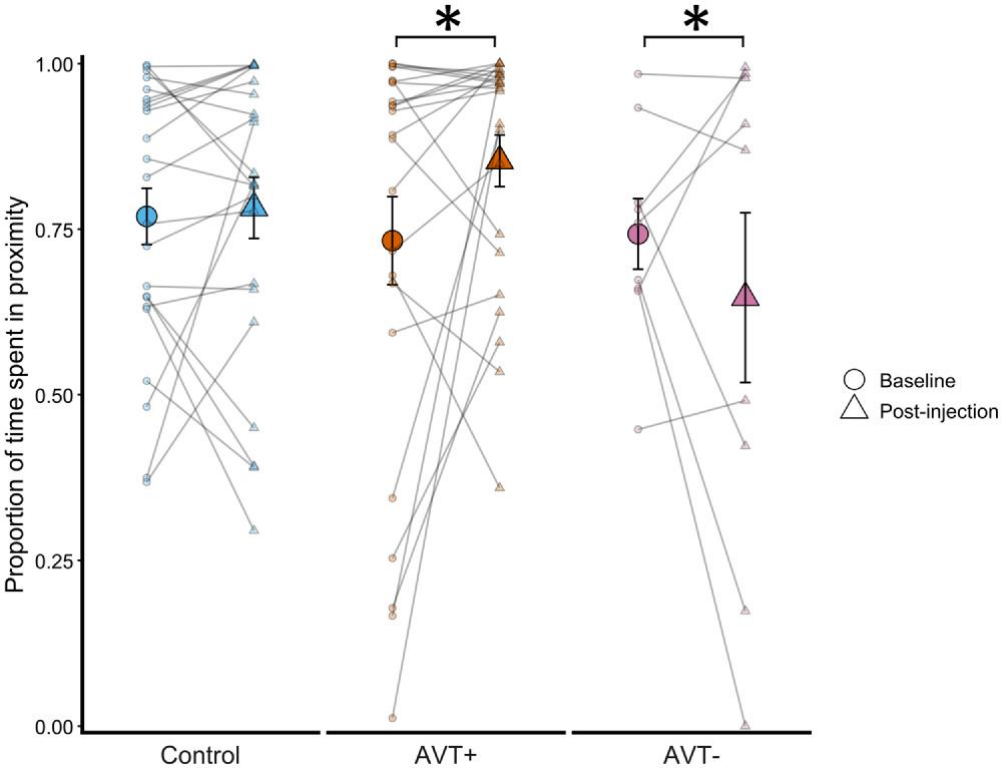
Short-term effects of vasotocin (AVT) on mother–offspring association, modeled as proportion of observed time that mother and offspring pairs spent in proximity (<20 cm) in both the baseline and the post-injection periods. Larger shapes and error bars represent mean ± standard error, and small shapes connected by lines represent paired individual data. Asterisks indicate significant differences. We found that AVT supplementation increased time spent in proximity, and blocking AVT reduced time spent in proximity.

When analyzing the data from only the baseline observations, we found no difference in the proportion of observed time spent in proximity (within 20 cm) between the 3 treatment groups (Material). However, comparing the baseline to the post-injection observations revealed effects of both AVT administration and receptor antagonism on adult–offspring proximity (*R*^2^ = 0.99, *f*^2^ = 0.04, Table 2). Specifically, we found a significant negative interaction between assay and proportion of time in proximity in the AVT− treatment group (Est = −0.46, *P* < 0.001), and a significant positive interaction between assay and the time spent in proximity in the AVT+ treatment group (Est = 1.02, *P* < 0.001) (Fig. 2). When examining post hoc pairwise comparisons, we also found a marginally significant difference between the AVT+ and AVT− groups in the post-injection observations (Est = 1.72, *P* = 0.051). The full model provided significantly better fit than the null model (DHARMA simulated LRT, *P* = <0.001).

**Table 2:** Model output from GLMMs analyzing behavioral responses under different AVT manipulations.

Model	Comparison	Fixed effect	Estimate	Standard error	*P* value
Short-term proximity	NA	Treatment (vasotocin)	−0.08	0.35	0.88
		Treatment (manning)	−0.33	0.51	0.62
		Assay (post-injection)	−0.005	0.66	0.31
		Vasotocin × post-injection	1.01	0.005	<0.001*
		Manning × post-injection	−0.46	0.009	<0.001*
Long-term proximity	Mid vs distant	Treatment (vasotocin)	0.31	0.35	0.37
		Treatment (manning)	−0.14	0.49	0.78
		Day	−36	0.29	<0.001*
		Vasotocin × day	−0.02	0.02	0.36
		Manning × day	−0.01	0.02	0.53
	Proximity vs distant	Treatment (vasotocin)	0.97	0.36	0.006*
		Treatment (manning)	0	0.5	1
		Day	0.04	0.01	<0.001*
		Vasotocin × day	−0.05	0.02	0.004*
		Manning × day	−0.02	0.02	0.45
Maternal aggression	NA	Treatment (vasotocin)	−0.69	0.49	0.16
		Treatment (manning)	0.13	0.47	0.79
		Assay (post-injection)	0.14	0.11	0.23
		Mothers’ temperature	0.29	0.08	<0.001*
		Mother’s SVL	−0.01	0.19	0.97
		Original treatment (vasotocin)	0.79	0.41	0.06
		Original treatment (manning)	0.09	0.57	0.87
		Vasotocin × post-injection	−0.15	0.22	0.49
		Manning × post-injection	−0.2	0.18	0.25

ID was included as a random effect in each model. Significant *P* values (<0.05) are marked with an asterisk. Comparison column only relevant for the multinomial mixed model.

**Figure 3:**
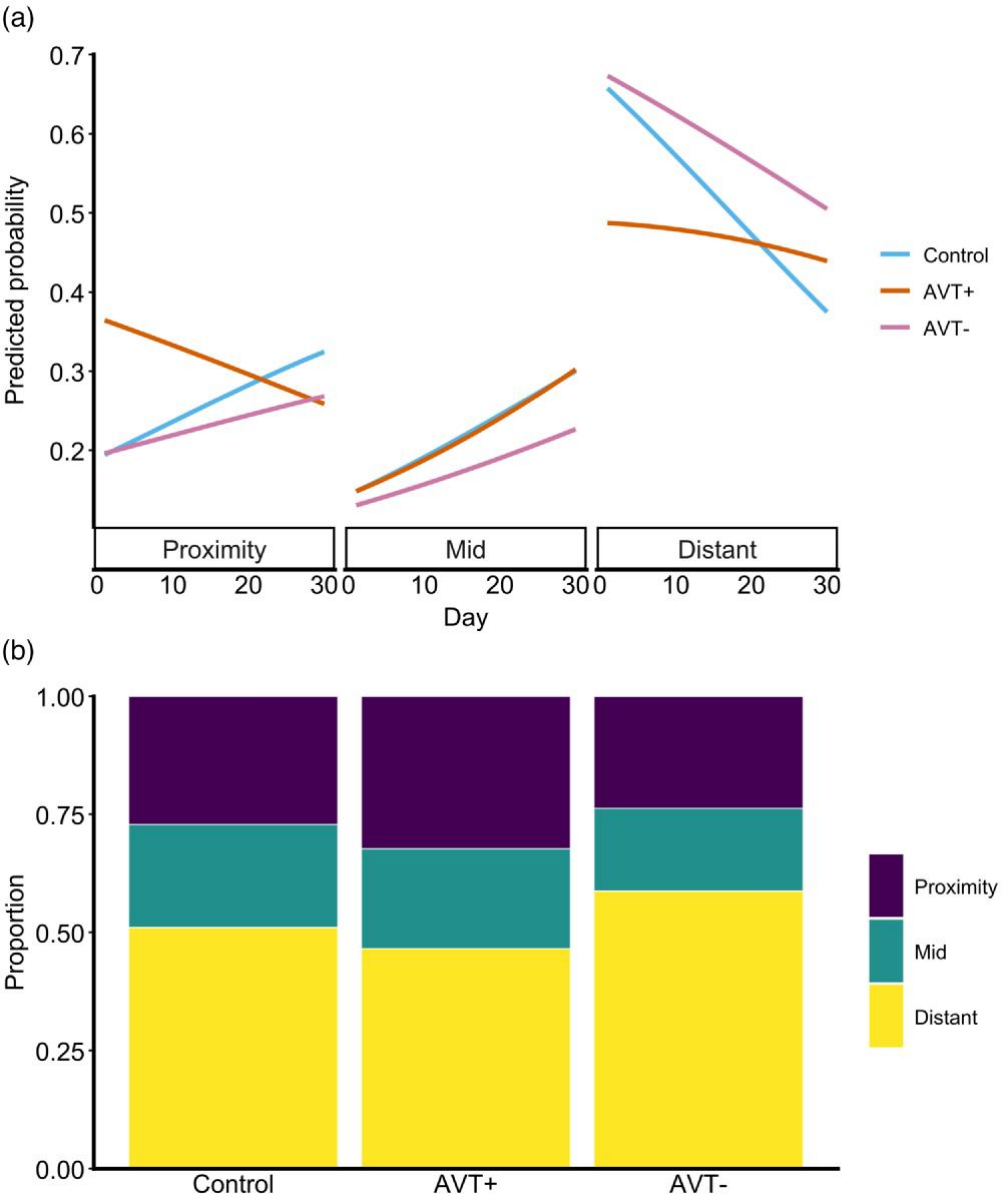
Long-term effects of vasotocin (AVT) on mother–offspring association. a) Predicted probabilities extracted from a multinomial mixed-effects model show how AVT supplementation increases the likelihood of mother–offspring pairs being found in “proximity”, and that this increased probability decreases over time until it converges with the probability for the control group at approximately day 20. b) Raw data displayed as relative positions of mother–offspring pairs as a proportion of total observations per treatment group, across the entire 30-d observation period.

Examining the long-term effects of the AVT manipulations, we found a significant effect of the AVT+ treatment on the likelihood of these lizards being found in close proximity to each other compared with being found far from each other (Est = 0.97, *P* = 0.006; Table 2). This represents a significant effect of AVT supplementation at a hypothetical observation day 0, though this nevertheless represents a long-term effect as day 1 of measurement was still 24 d post-injection. We also found a significant negative interaction between AVT+ and observation day (Est = −0.05, *P* = 0.004), suggesting that the effect of AVT supplementation slowly diminished over time. To better understand this relationship, we extracted the predicted probabilities from our multinomial mixed-effects model and plotted them over time (Fig. 3a). These predictions indicate that by approximately day 20, the probability of AVT+ lizards being found in the proximity category converges with that of the control group. We also found a significant negative effect of day on likelihood of finding lizards in the proximity (Est = 0.04, *P* < 0.001) or the mid-range (Est = 0.04, *P* < 0.001) categories when compared with the distant category, suggesting that in the control group, lizards were found closer to each other over time. Model comparison using AIC indicated that our fixed effects improved model fit compared with the null model (ΔAIC = 13.7). We found no significant effect of Manning compound, though the raw data do suggest a trend for AVT− lizards to be found in the “distant” category more often than the other treatments (Fig. 3b).

### Effects of AVT on protective maternal aggression

**Figure 4:**
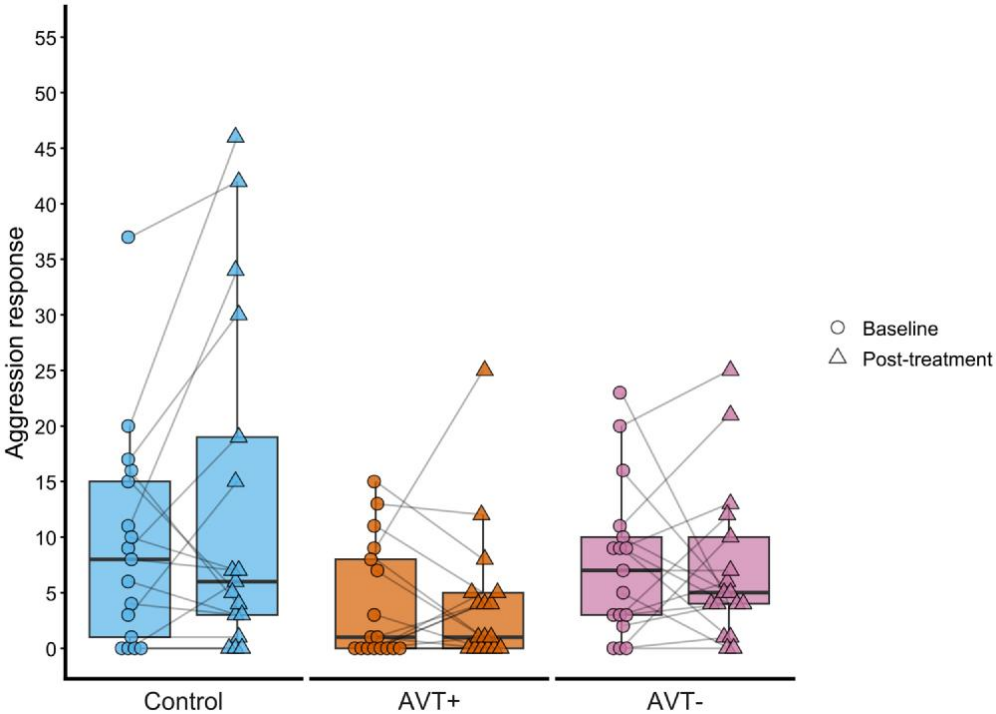
Effect of vasotocin (AVT) on maternal aggression in both baseline and post-treatment territorial intrusion assays for each of the 3 treatment groups (*n* = 17 per treatment). Connected points represent individual lizard’s aggression response score (summed values of bite, arch and gape). No differences were found between the baseline and post-injection assays.

We compared aggression response (the sum of bites, back arches, and gapes) across our 3 pharmacological treatments, both before and after injections (*R*^2^ = 0.95, Table 2). We found no significant effect of pharmacological treatment on maternal aggression (ANOVA; χ2(2) = 3.60, *P* = 0.17) or on the interaction of treatment group and assay (ANOVA; χ^2^(2) = 1.44, *P* = 0.49; Fig. 4). There was also no significant effect of either mothers’ SVL (Est = −0.01, *P* = 0.97) or original pharmacological treatment (Anova; χ^2^(2) = 3.95, *P* = 0.144) on aggression. We did find a significant positive effect of a mothers’ temperature on aggression response (*f*^2^ = 0.05, Est = 0.29, *P* < 0.001; Figure S3). The full model provided a significantly better fit than the null model (DHARMA simulated LRT, *P* = 0.016). Repeatability in an individual’s aggression response was reasonably high at 0.63 (95% CI: 0.43 to 0.77) as determined using an intraclass correlation coefficient.

## Discussion

We experimentally tested the role of AVT in mediating parental behavior in the facultatively family-living lizard, *L. whitii*. We gathered data on both mother–offspring proximity, in the short- and long-term, as well as data on maternal aggression in response to a territorial intruder. We found that AVT supplementation increased mother–offspring proximity and blocking AVT reduced proximity in the short-term only. Conversely, AVT manipulations appeared to have no effect on maternal aggression. The implications of these results in the context of social evolution are discussed below.

The AVT agonist and antagonist treatment caused a corresponding, short-term, increase or decrease in time spent in proximity between mother and offspring in the 90 min following injection. These results are consistent with both of our predictions, as well as with the only other study to test the role of AVT in reptilian maternal behavior (Lind et al. 2017). Specifically, rattlesnakes given Manning compound spent significantly less time in proximity with their offspring compared with those given saline (Lind et al. 2017). Our results are also consistent with work in rodents (Pedersen et al. 1982; Wang et al. 1994; Bosch and Neumann 2008), primates (Taylor and French 2015), and birds (Klatt and Goodson 2013) demonstrating the role of AVT in mediating parental behavior. Combined, these results suggest that vasotocin has a conserved role in mediating parental behavior that spans vertebrates, including lizards.

Our findings indicate that the role of AVT in shaping parental behavior may extend well beyond immediate behavioral responses. A single injection of AVT resulted in increased mother–offspring proximity 3 wk later. This effect slowly diminished across our observation period and converged with the results from the control group at approximately day 20. Although the effect sizes were modest and the sample size relatively small, the pattern is consistent with the idea that AVT-mediated changes can influence social outcomes over extended periods (see also Boer et al. 1994; Stribley and Carter 1999; Baran et al. 2016). Although we show that AVT itself clears rapidly from circulation, downstream physiological responses can persist for days, as shown in *Zootoca vivipara* (Meylan et al. 2017), and lingering behavioral effects of antagonists such as Manning compound have been observed in other taxa (Lind et al. 2017), although never for such an extended period as observed here. We also found that in the control treatment, mother–offspring pairs were more likely to be found in proximity to each other later in the observation period. Although this might initially suggest that mothers are becoming more tolerant over time, we believe that this effect is an artifact of our experimental design. Lizards were moved outdoors into large semi-natural enclosures for this observation period, and although they were given 24 h to acclimatize to these new conditions, the increased distance between pairs early in the observation period may represent increased exploration. This artifact may also be obscuring some long-term effects of Manning compound, which appears to increase the likelihood of lizards being found distant from each other, though this effect is not statistically significant. Repeating this experiment with consistent natural housing conditions may reveal these effects more clearly.

The exact mechanisms facilitating these longer-term behavioral effects are unclear, but 1 possibility is that behavioral feedback loops triggered by initial AVT-induced increases in proximity may sustain elevated AVT activity via social contact and touch (Griesser et al. 2025). It is also possible that learning plays a role in maintaining these long-term effects. For example, after a social defeat, mice will avoid their aggressor for up to 15 d, even in the absence of further aggression (Qi et al. 2018). Similar processes may be at play here, where Manning compound induced lack of tolerance from the mother may appear to persist because her offspring has learnt to avoid a nontolerant (and therefore potentially dangerous) conspecific. Growing evidence demonstrates that AVT can have long-lasting developmental impacts, including on emotionality, social attachment, memory, and aggression (Boer et al. 1994; Stribley and Carter 1999; Baran et al. 2016; Simmons et al. 2017; Finton and Ophir 2022). These effects are referred to as organizational, meaning they occur during critical periods of development and cause long-lasting changes that lay the groundwork for future behaviors (Phoenix et al. 1959). These studies all focus on the organizational effects of hormones when manipulated early in development, but the transition to motherhood itself constitutes a hormonally sensitive period, which can last for weeks postpartum (Cárdenas et al. 2020; Orchard et al. 2023). Similarly, these studies involved chronic administration of hormone agonists or antagonists, and to our knowledge no previous work has investigated the long-term effects of a single dose of AVT or an AVT antagonist. Interestingly, however, a single dose of the closely related nonapeptide oxytocin can produce behavioral changes months later (Noonan et al. 1989). Although the effects in the current study did diminish over time, these lines of evidence suggest that exogenous AVT delivered during this maternal critical period may have the capacity to produce enduring, potentially organizational effects on parent–offspring associations—a possibility that warrants further investigation.

In contrast to our results on mother–offspring proximity, we found no effect of AVT manipulation on female aggression (sum of bites, gapes, and arches). The lack of an observed effect here may reflect a dose-dependent response to our treatments. For example, in damselfish, it was shown that low and high doses of AVT caused no difference in territorial aggression, but mid-range doses caused large increases in overt aggressive behavior (Santangelo and Bass 2006). However, given the clear effect of our treatments on mother–offspring associations, we feel the results suggest that AVT does not mediate aggression in female *L. whitii*. This contrasts with previous studies, which have found strong effects of AVT on aggression-like behaviors in lizards (Dunham and Wilczynski 2014), fishes (Santangelo and Bass 2006), birds (Goodson and Adkins-Regan 1999) and mammals (Bosch and Neumann 2012). It is thought that *L. whitii* achieves family living through inhibiting aggression toward their own offspring whilst maintaining it toward other individuals, thus allowing the offspring to benefit from remaining on the parent’s territory. It is plausible that AVT has been de-coupled from its usual role as a mediator of aggression in this context, because of the role it plays in parent–offspring associations. While other studies have found that territorial species become less aggressive under AVT exposure (Goodson 1998; Semsar et al. 2001; Backström and Winberg 2009), this may be counter-productive in *L. whitii* as aggression also functions to protect offspring from infanticidal conspecifics (Sinn et al. 2008). Conversely, if AVT increased aggression in *L. whitii* (as it does in nonterritorial species; Goodson 1998; Goodson and Adkins-Regan 1999), this could lead to maladaptive misdirected aggression toward their own offspring (or social partner). Theory predicts that protective aggression should be linked to both higher rates of central AVT and testosterone release (Van Anders et al. 2011). And yet, testosterone also does not predict aggressive behavior in female *L. whitii* (While et al. 2010), further supporting the idea that this pathway has been de-coupled (or perhaps not yet co-opted) into mediating aggression in this context. Other species maintain testosterone-independent territorial aggression outside the breeding season, possibly mediated via melatonin (Demas et al. 2023), and perhaps *L. whitii* also display seasonal differences in the mechanisms controlling aggression.

The observed effects of our AVT manipulations may carry important fitness implications for offspring if increased proximity to a parent has functional consequences. There are several contexts in which closer parent–offspring association could be beneficial, including reduced predation risk (O’Connor and Shine 2004; Sinn et al. 2008), enhanced access to high-quality resources such as optimal basking or foraging sites (Botterill-James et al. 2016), and exposure to a stable social environment that can shape behavioral development (Munch et al. 2018). Importantly, such benefits could feed back to influence the evolution of social behavior more broadly. Prior work shows that ecological factors—such as clustered habitat or high population density—promote parent–offspring associations (Botterill-James et al. 2016; Halliwell et al. 2017), and that the maintenance of these associations is likely shaped by variation in parental tolerance. If our exogenous AVT treatments mimic natural variation in endogenous AVT release, then differences among adults in AVT dynamics may contribute to the observed variation in parental tolerance (eg, Russell et al., in prep; Fig. 2). Under environmental conditions where nonapeptide-driven changes to the social environment confer fitness benefits, even small shifts in AVT release—and the resulting shifts in social bonding—could provide a mechanistic pathway through which solitary species begin the transition toward family living (eg, Griesser et al. 2025).

Finally, although we saw a clear effect of our treatment on parent–offspring associations, the exact mechanism of action through which our AVT manipulations influence behavior is unclear. The extent to which peripheral AVT can cross the blood–brain barrier is debated (Mens et al. 1993; Yamamoto et al. 2019), though it likely that only small quantities of AVT would need to cross the barrier in order to have an appreciable effect (Kang and Park 2000). Nevertheless, we may have seen different, or more salient shifts in behavior had we administered our manipulations centrally, and future work should seek to directly compare these delivery methods (eg Moore and Miller 1983; Goodson et al. 2009). Our study showed that the nonapeptide AVT is involved in mediating the simple associative relationship between mother and offspring that characterizes parental behavior in *L. whitii*, and that small changes in peripheral AVT can have long-lasting impacts. This is broadly consistent with the literature, but the finding that AVT does not mediate aggression suggests that its patterns of action in this species may not translate to other taxa. Aggression in *L. whitii* is necessary to protect territories, but it must be carefully directed so as not to result in infanticide. Different hormonal mechanisms may more subtly mediate this behavior to facilitate the unique life history of these lizards. Investigating the effects of nonapeptides and other hormones in a range of organisms, particularly in “simple” social systems, will further shed light on the evolutionary history of such proximate mechanisms as drivers of social evolution.

## Supplementary Material

arag082_Supplementary_Data

## Data Availability

Analyses reported in this article can be reproduced using the data provided by Lake et al. (2026).
