# Supplementary material for: Vasotocin influences mother–offspring associations in a facultatively family-living lizard
Source: Behav Ecol. 2026 Jul 21;37(5):arag082. doi: 10.1093/beheco/arag082 (PMC13448473; doi:10.1093/beheco/arag082)
Supplement: Supplementary file 1 [file arag082_supplementary_data.zip › arag082_Supplementary_Data.zip!/Supplementary Material for Whitii AVT Manuscript.docx]

**Supplementary Material** **for - Vasotocin influences mother-offspring associations in a facultatively family-living lizard**

**Supplementary analyses**

*Short term mother-offspring associations*

In the mother-offspring association observations, mothers were randomly assigned to receive either an injection of AVT (AVT+), manning compound (AVT-), or a saline control. To test whether there were any differences in mother-offspring association between these groups before we conducted our manipulations, we fitted a model using only the baseline data. We also wanted to test whether variation in the offspring traits had an influence on the mother-offspring associations. We chose to include offspring age, weight at birth, and snout-vent length (SVL) at birth as potentially confounding variables. As the latter two are tightly linked, we ran a principal component analysis to extract a single variable for these data. Weight and SVL loaded tightly onto a single principal component (PC1), which explained 73% of the variance in the data.

As in the main text, proportion of time in proximity was fitted with a binomial distribution against pharmacological treatment using the *lme4* package. PC1 and offspring age in days were both included as fixed effects, and ID was included as a random effect. Model fit was assessed with the *DHARMa* package. We found no significant effect of treatment in the baseline data, and no significant effect of either offspring age or PC1, which represented SVL and weight at birth. The full model did not provide a better fit than the null model (simulated likelihood ratio test, P > 0.05), indicating the fixed effects did not explain variation in mother-offspring proximity. Model output is displayed in table S1.

*Maternal Aggression*

To test whether offspring age impacted the aggressive response of mothers toward a simulated territorial intrusion, we fitted an additional model using only the baseline data from the maternal aggression trials. We analysed differences in aggression response (sum of bite, gape and arch behaviours) across offspring age in days using a GLMM fitted with a negative binomial distribution. Offspring age was scale-transformed, and mother’s ID was included as a random effect. As in the model described in the main text, we included the log of trial time as an offset in our model because each aggression assay was variable in the time it lasted; this was because lizards were allowed to flee and thus end the trial. We found no significant effect of offspring age on aggression response. Model fit was assessed with the *DHARMa* package. The full model did not provide a better fit than the null model (simulated likelihood ratio test, P > 0.05). Model output is displayed in table S1.

**Table S1**. Model output for supplementary GLMMs. ID was included as a random effect in each model.

| Model | Fixed Effect | Estimate | Standard Error | P Value |
| --- | --- | --- | --- | --- |
| Short-term proximity (Baseline data only) | Treatment (Vasotocin) | 0.44 | 2.27 | 0.51 |
|  | Treatment (Manning) | -0.41 | 1.00 | 0.67 |
|  | Offspring age | 0.04 | 0.24 | 0.88 |
|  | PC1 (weight x SVL) | -0.25 | 0.29 | 0.4 |
| Aggression x offspring age | Offspring age | -0.36 | 0.23 | 0.13 |

**Supplementary figures**

**Figure S1**. 3D printed lizard model used in the maternal aggression trials to simulate a territorial intrusion. Model had a snout-vent length of approx. 77mm

**Figure S2**. Concentration (mean ± SEM) of AVT in the plasma of *L. whitii* after an intraperitoneal injection of AVT. Lizards were sampled at 0.5-, 1-, 2-, 6-, 12-, 24-, and 48-hours post injection. The purple dashed line indicates average baseline AVT concentration estimated from control samples in the parallelism and quantitative recovery assays (~1280 pg/ml). Measured concentrations of AVT dropping below the estimated baseline concentration may represent negative feedback mechanisms.

**Figure S3.** The effect of a lizard’s temperature on aggression response in the maternal aggression trials. Each dot represents an individual lizard’s aggression response score (summed values of bite, arch and gape).
